# Discussion on Erysipelas*Montreal Medico-Chirurgical Society, from Montreal Medical Journal.

**Published:** 1895-09

**Authors:** 


### Abstracts and Selections.

### Discussion on Erysipelas.*

Dr. F. W. Campbell said that an old friend of his always got an attack of erysipelas when he exposed himself to the north wind. He mentioned a few things in the way of local treatment which had not been referred to by Dr. Roddick, namely, lead, arnica and opium; and a form used by the late Dr. Crawford, of this city, the application of tincture of iodine, and subsequent dusting of the part with flour to protect it from the atmosphere. He thought he had the power of limiting the diesase by the application of the solid stick of nitrate of silver, although it occasionally failed. A local application of the liquid extract of ergot contracted the vessels over the part; an illustration of which had occurred to him during the last ten days. A child burned its big toe, it was neglected, and erysipelas set in, spreading up as far as the knee, it passed the knee, got as far as the hip, and was extending up the back. Ergot was applied at night, with direction to repeat in the morning, but in the morning it was not required,—the skin was shriveled and dry. Dr. Roddick had approved of calomel, and gave 5 to 10 grains. He (Dr. Campbell) often prescribed 10 grains of calomel to a child 10 months old, and the result was only two or three medium evacuations.

*Montreal Medico-Chirurgical Society, from Montreal Medical Journal.

Dr. Bell said the more one considers the subject, the more one is confirmed in the opinion that the disease erysipelas is dealt with in a most empirical manner. I am glad to hear Dr. Adami say that he believes, and that many bacteriologists believe that erysipelas is only one of the manifestations of the presence in an active condition of the streptococcus. I think it quite right to look upon cutaneous erysipelas as a specific condition. Such cases are comparatively easy to deal with. There can be no question as to the advisability of isolating such cases; but when we come to deal with cases not quite so typical, when we come to deal with cases where the lymphatics beneath the skin are affected, we can not fail to ask ourselves the question, what is the difference between this disease, erysipelas, and the lymphangitis which attacks the dissecting room student, the surgeon, or the butcher? And why should we insist upon isolating one form of streptococcus disease, that called erysipelas, and not isolate other inflammatory conditions which we attribute to the same coccus? The series of cases mentioned by Dr. Adami as having occurred in the Royal Victoria Hospital under my observation, have already been reported by me to this society. Another series of cases, reported to the Montreal branch of the British Medical Association about a year ago, was as follows: A woman recently confined by a midwife in a district where scarlatina prevailed, died of purulent peritonitis, and a house-surgeon and a student became infected from her in the performance of their separate duties in the ward and post-mortem rooms, the one contracting an erysipelas, the other a subcutaneous lymphangitis. Then, again, there is a class of cases which I have several times observed: In cases of appendicitis ending in abscess, which makes its exit from the peritoneal cavity, either furrowing along the psoas muscle or in the tissues of the abdominal wall, it frequently happens that when such cases are operated upon and the abscess drained, that during convalescence the patient is attacked by erysipelas, which is again followed by pyæmia. Br. Bell then mentioned the particulars of a case of this kind, which, when the abscess was opened and drained, was followed, in about three weeks, by erysipelas about the wound, afterwards involving the face and finally ending in pyæmia. Another old patient, admitted to hospital on February 1st, 1895, was operated upon February 11th, and did very well after the operation. On the 28th of March, pneumonia set in, followed on April 7th by erysipelas of the face. Later on, he developed isolated patches of erysipelas over the abdomen. On the 20th of April (after the chest had cleared up and he seemed quite recovered from his pneumonia) he was isolated, but an empyæmia developed, and he died on April 25th. Now, when erysipelas is clinically interchangeable, with these other conditions, I do not see how we can look upon it as a specific disease. I think the latter view is based upon observations of too narrow a scope, that it is a premature conclusion, drawn from insufficient data, and that the theory expressed by Dr. Adami, that erysipelas is only one of the evidences of the pathogenic qualities of the streptococcus, will be found to be the more correct one. Compare this subject with our knowledge, past and present, of tuberculosis and tuberculous conditions. For instance, "white swelling of the knee" and scrofula, conditions which, a few years ago, it would have been ridiculous to regard as similar to pulmonary phthisis, are all to-day known to be manifestations of the same disease--tuberculosis. So I think in time we will come to regard all these diseases above mentioned (erysipelas, pyæmia, etc.), with others, such as œdema glottidis, angina Ludovici, and many of the so-called surgical or septic fevers, as distinct manifestations of the effects of some micrococcus (streptococcus) alone, or in connection with the other micrococci (the staphylocci, etc.) I will just mention here the clinical history of three cases of erysipelas which we had recently in the Royal Victoria Hospital. First, on the 7th of April, the old man already referred to developed erysipelas. On April 4th, there was admitted a man from the country, who had a week or ten days previously injured his hand, with the development of cellulo-cutaneous erysipelas of hand. Within a day or two he developed metastatic abscess, or a typical pyæmia, with abscesses in the joints of the left hand. About the same time, Dr. Buller did a plastic operation upon the nose of a patient in the same ward. Dr. Buller's patient was admitted on the 1st of February, operated upon on the 10th of April, and the onset of erysipelas on the 11th of April. Here this case of erysipelas was admitted on the 4th, the old man developed erysipelas on the 7th, and Dr. Buller's patient on the 11th; now, just exactly which of these patients is responsible for introducing the disease, is a problem. I am inclined to think, from considering these appendix cases, and the remarks made by Dr. Adami, that the old appendix case was the cause of inoculating Dr. Buller's patient. Then again, in speaking of the interchangeability of this disease, I forgot to mention the puerperal period. Dr. Baker, of the veterinary school, on Sunday night, in delivering a cow which had been in labor for more than 24 hours and roughly handled by stablemen, noticed the animal's vagina in a swollen condition. In using a hook instrument for delivery, he scratched the back of his hand; I saw it the next day. On Thursday night, I incised his hand freely, not that there was any very great indication for it, except to relieve pain. That same night, without any pressure being applied to the finger, it became black, gangrene set in, and extended rapidly up the finger. The gangrene, however, was arrested, but the finger had to be amputated. Here, then, is a case of apparently rapid infection from the mucous membrane of the cow's vagina prior to delivery, and therefore not a condition of puerperal fever, and was only brought about by bad management or unclean operators. Now, everyone admits that the ordinary cutaneous and cellulo-cutaneous erysipelas is contagious, but I am inclined to think that the contagiousness of erysipelas is not so great as is generally believed. On the other hand, the deeper lymphangitis, and many other forms of the so-called septic fevers, perhaps all of them, in which danger of contagiousness is not recognized at all, may, under certain conditions, be contagious, and possibly the difference between the contagiousness of erysipelas and these other conditions is only a difference in degree, and yet not nearly so great as is generally thought. As to treatment, I can not say that I have any definite views as to what is the proper medicinal treatment at all. Of course the local surgical treatment and ordinary antiseptic treatment, so far as it can be carried out, is safe and rational.

Dr. England wished to express his appreciation of the theory upheld by Dr. Adami. Everyone who had had a general practice, and thus an opportunity of seeing general septic conditions, including among them erysipelas, would see the *rationale* of the theory he presented. He could call to mind at least three or four similar cases to those narrated by Dr. Bell—namely, a septic wound to be followed by a pneumonia, then by facial erysipelas, and finally ending in pyæmia and death. All these conditions arising from a simple abrasion of the skin. So also, between puerperal fever and erysipelas a relationship exists, so much so that careful physicians gave up accouchement work when attending erysipelas. The streptococcus of erysipelas introduced through abrasions in the vagina into the puerperal woman, will set up a most severe, and generally fatal general septic infection,—a progressive sepsis without erysipelas, about the genitals, showing itself. From his clinical experience, this definition given to the disease by Dr. Adami, not limiting the imflammatory process to an inflammation of the skin and mucous membranes, but considering it in a wider sense, seemed more rational. He further mentioned that he had seen two cases of facial erysipelas run an ordinary course of moderate severity, although occurring during the first week after delivery, without any special symptoms bearing upon the generative tract.

Dr. Buller, speaking in regard to the case referred to by Dr. Bell, in which erysipelas developed after a plastic operation, said that the first operation in this case had been an extensive one, and was followed by no bad results; the second operation had not been so extensive, and although performed with all the usual antiseptic precautions, erysipelas followed. He looked upon the nasal organ as especially liable to take on erysipelas action. This man, referred to, must have inspired the erysipelas poison; there was no sign of erysipelas in the wound itself, but the skin of the nose, quite apart from the wound, was the first place in which the disease made its appearance. It was a well known fact that people who had any slight solution of continuity in the nasal mucous membrane, were very liable to take erysipelas. Another point in this connection was that one occasionally met with erysipelas of the face in which the disease extended to the orbit, after the manner of deep-seated or phlegmonous erysipelas, and caused pressure upon the eyeball and optic nerve, commonly ending in blindness. In this case, he had excavated considerably into the orbit, and exposed the orbital tissues pretty freely; but although the erysipelas spread over the region of the wound, it did not enter the orbit. He thought this was an indication that there might possibly be some difference between the germ action which was necessary to set up a deep erysipelas, and the germ action which sets up the cutaneous variety. Although this man seemed to have contracted his erysipelas from the deep variety of the disease, the kind he had was cutaneous, and he was not affected by the deep kind. Perhaps he had no special susceptibility for the deep erysipelatous action, and therefore it was impossible for him to contract it. In reference to erysipelas in the different seasons of the year, Dr. Buller met with most of his cases in spring and autumn. Whatever may be the cause, it must be very widespread; erysipelas occurred in houses where the individuals were perfectly healthy, one member catching it and the others entirely escaping. Some time ago, he saw a child who had erysipelas following a slight ulceration of the margin of the lid; it came into contact with no other case of the affection, and therefore must have received the poison through the atmosphere. Whatever the poison was, it must be very widespread, else the sporadic cases could not be accounted for.

The President, referring to the remarks of Dr. Roddick as to hot, moist applications being advisable, and Dr. F. W. Campbell's recommendation of dry application in the form of flour, said he remembered, some years ago, in 1854, in Saint George's Hospital, a large number of cases of erysipelas occurred, in fact, the disease followed almost every operation, until at length the authorities were forced to close up the infected wards. It had been the custom to have these wards washed out every morning. They now, after closing them, caused the walls and floors to be freshly painted and dry rubbed. They were then opened, and no more cases of erysipelas occurred. It was impossible to say positively that the moisture, evolved by the daily washing, had the effect of keeping up the affection; but he thought it likely that it had a tendency that way, as moisture was one of the conditions of growth of bacilli, and it was possible that it should also have some influence in the treatment. The manner in which the disease extends, leaving the central part and spreading around, reminds one of what was seen in fields in the shape of the "fairy rings"—always growing larger and larger, but never growing on the same spot a second time. It may be possible that this streptococcus, like the fungus of the fairy ring, after growing a certain while produces something which prevented its developing any further in that particular spot.

Dr. Adami.—Dr. Mills wished to know if other germs could be excluded as producers of erysipelas. We can occasionally get slight reddening in connection with germs that are not chain cocci. For instance, in cases of ordinary abscess, one gets a certain zone of reddening, and apparently cutaneous and lymphatic disturbance in the region of the abscess. One, therefore, can get a certain blush in the neighborhood of an abscess caused by the pyococcus. This is about all that can be said on the matter.

With regard to Dr. Buller's case, of want of extension to the parts deep in the orbit, I will point out that by cutting across the lymphatics he may have interrupted the flow of lymphanoids.

The cocci are themselves immovable, and are either conveyed by direct growth in stagnant fluid or along currents, not to any extent against those currents. Possibly extension would, in any case, be less likely to occur deeply. I only throw this out as a suggestion.

I can not agree with Dr. Roddick that the streptococcus of erysipelas is specific. I will admit that it is peculiar; but not that it is specific. I think we are now on the eve of regarding the series of suppurative diseases due to the chain coccus in the same light as we do now regard the whole group of diseases due to tuberculosis. Some years ago one would be regarded as foolhardy who stated that white swelling of the knee, or lupus, were not specific and distinct diseases.

What has been said with reference to the iron treatment of erysipelas reminds me vividly of an incident while I was still "walking the hospital" at Manchester. I was making the rounds with one of the surgeons, and he came to a stand before a convalescent patient. "Gentlemen," said he, "let me once more impress upon you that tincture of the perchloride of iron is a specific for erysipelas. You see here my fortieth case in succession treated thus without a single death." One of the physicians who had been called in to examine a patient for admission to the medical side happening to hear the statement, came across the ward at this, and beckoning the surgeon to one side, said to him sufficiently loud for me to hear: "My dear W——, what an odd coincidence! I had begun to doubt the efficiency of iron, and have been treating erysipelas with simple rest in bed and aperient. I've just had forty cases also on this treatment—without a death!"

The treatment mentioned by Dr. Campbell of ringing or delimiting the advancing margin by the application of lunar caustic is undoubtedly sometimes effective, and it may be worth while here to give Sims Woodhead's explanation of the *rationale* of the process. As I said before, there are in the reddened erysipelatous area numerous leucocytes together with destruction of the cocci; in the zone outside this one finds cocci without any excess of leucocytes. The application of the caustic in this outer zone sets up a simple inflammation with migration of leucocytes and presumable destruction of the cocci. I would suggest that, bearing in mind the fact that chain cocci may be found more than an inch beyond the reddened margin, the line of application should be painted at least one inch and a half outside that margin, thus enclosing the living, active cocci between two barriers of typical inflammation.

What I have said previously concerning saprophytic existence of the streptococci on the skin and mucous membranes is not peculiar to this one organism. Very numerous observers have similarly discovered the pneumococcus, the diphtheria bacillus, the bacillus pyocyaneus, and yet other organisms capable of producing well-marked lesions within the tissues of the body upon the skin and mucosæ of those that are perfectly healthy. In fact, the diplococcus of pneumonia has been found by Sternberg as an inhabitant of his sputum for months at a time, and this is true for the sputum of 15 per cent more of healthy individuals.

Dr. Hingston, in reply, said the tendency of the best medical minds to-day is to synthetize rather than to analyze; to group together diseases having features of affinity, rather than to separate and create new diseases which have certain lineaments or single parts which seem to be different from the general outline. I am inclined to think there are many in this room who will live to see diseases grouped together on a large scale—very large groups brought under one head, like those groupings of Erasmus Wilson in skin diseases, where he reduced the whole of them, with all their chief distinctions, to three or four heads, for therapeutic purposes. And thus it may be that erysipelas may come to be considered as simply a form of inflammatory action modified by conditions proper to the patient, either in his constitution or in his surroundings. I have had some personal experience of erysipelas in my own person; it has, in fact, been my *bête noir*, having had any number of attacks. The worst one was from a little scratch of a razor. As soon as I was dressed I started off on a long journey in winter time. The weather was extremely cold. I traveled incessantly, changing horses three times. As a cold wind was blowing from the north I must say, if erysipelas arises from a living bacterial principle outside of the body, it must have taken up its abode on this occasion in the otherwise pure north wind, and have pounced down upon the razor scratch like a hawk upon its quarry, as I had symptoms of the disease before the end of the journey. If the microbe crawled up from his habitat in the mouth or nostril, then must its power of resisting existing cold have been very great. On another occasion, on removing my eye-glass, I made a little scratch, which was followed by an attack of the disease. The beak of a bird, on another occasion, brought on this trouble. With many attacks of this affection arising in a variety of ways, I have had some little experience as to the treatment and as to the comfort afforded by different local applications. I have tried many things which either my friends or my own experience could suggest. Unctuous applications afforded no relief; the old fashioned flour gave most ease: but the suspicion crossed my mind that it was not the flour, but the firm yet gentle mechanical pressure with which it was applied, that gave relief. The application of a little soft rag without the flour gave me the same amount of relief and for an equal length of time.

As to the question of the contagiousness of erysipelas, some seem to think the matter settled. Whether so or not, the most prudent thing is to act as if it were highly contagious, and not to put patients, after an operation, in the neighborhood of those who have or who have had erysipelas. In reference to treatment, two gentlemen spoke of the value of certain purgatives. I have a very strong feeling which I can not emphasize too forcibly, that purgatives should not be given in erysipelas. A dose of castor oil may be given at the beginning as a laxative, but not afterwards. We know that in certain fevers the exhibition of purgatives is objectionable. I have known again and again erysipelas to return when purgatives have been taken after the disease had seemingly subsided. The food should be nutritious and abundant, but as to liquid meats, such as broth and beef tea, I deny that they are foods at all. That, perhaps, may appear heretical to some, but on this question I am in harmony with an expression of opinion given before the British Medical Association some fifteen or more years ago, when the chairman of a committee, appointed the previous year to enquire into the value of liquid meats, gave it as the unanimous conclusion of the committee, after some crucial experiments, that liquid meats—or meats in suspension—not solution—are valueless as life or heat sustaining. On this question I share the opinion of the late Dr. Parker, of New York, when he said meat broths and meat teas and meat essences, when drank, are as useful to patients as their own urine. In conclusion, Dr. Hingston referred to Dr. Buller's proposition to plug the nares for the purpose of preventing the entrance of the streptococcus through that channel, and said he thought it would not be effective, as the connection existing between the buccal and nasal passages behind, would neutralize the desired effect when the patient was compelled to resort to mouth breathing.

